# The safety of patient management in family medicine in Slovenia during Covid-19: a cross-sectional study

**DOI:** 10.1186/s12875-023-02209-z

**Published:** 2023-11-29

**Authors:** Maja Cvetko Gomezelj, Špela Miroševič, Alina Verdnik Tajki, Ksenija Tušek Bunc, Esther Van Poel, Sara Willems, Zalika Klemenc-Ketiš

**Affiliations:** 1 Department of Family Medicine, Medical Faculty, University of Ljubljana, Ljubljana, Slovenia; 2 Department of Family Medicine, Medical Faculty, University of Maribor, Maribor, Slovenia; 3 Department of Public Health and Primary Care, Ghent University, Ghent, Belgium; 4 Ljubljana Community Health Centre, Ljubljana, Slovenia

**Keywords:** Covid-19, Family medicine, Patient safety, Patient management, Quality of care, PRICOV-19, Primary health care, General practice, Timeliness

## Abstract

**Background:**

During the Covid-19 pandemic, family medicine practices (FMPs) changed to improve safety against new coronavirus infections for both patients and employees. Protocols for treating patients with suspected Sars-Cov-2 infections were established to protect medical staff and other patients from being infected. However, these protocols also led to increased safety risks, such as delays in treating patients with other medical conditions. This exploratory study aimed to investigate safety risks in treating patients in FMPs during the Covid-19 pandemic and to suggest improvements to prevent Covid-19 in FMPs in Slovenia.

**Methods:**

A cross-sectional study was rolled out in FMPs in Slovenia as part of the international Pricov-19 study. Data collection on safety management during the Covid-19 pandemic in FMPs in Slovenia took place from November 2020 until January 2021 using a self-administered online survey for FP working in Slovenia. A chi-square test, ANOVA, independent samples t-test or bivariate correlation test was performed to explore associations regarding the safety of patients’ management variables.

**Results:**

From the 191 participating family physicians (FPs) (15.2% response rate), 54.8% reported having treated patients with fever (not Covid-19) late due to the new protocols at least once, and 54.8% reported patients with urgent conditions having been seen late at least once due to not coming. In the suburbs and rural environments FPs more often reported that at least once patient with a fever (not Covid-19) was seen late due to the protocol (*p* = 0.017) and more often reported that at least once patient with an urgent condition was seen late due to not coming to their FP (*p* = 0.017). The larger the practice, the more they reported that at least once a patient with fever (not Covid-19) was seen late due to the protocol (*p* = 0.012) and the more they reported at least once a patient with an urgent condition was seen late due to not coming to their FP (*p* = 0.012).

**Conclusion:**

Covid-19 affected the safety of patient management in FMP in Slovenia. The most common problem was foregone care. Therefor, protocols for chronic patient management in the event of epidemics need to be established.

**Supplementary Information:**

The online version contains supplementary material available at 10.1186/s12875-023-02209-z.

## Background

During the Covid-19 pandemic, work organization changed in family medicine practices (FMPs) in Slovenia in order to improve safety against new coronavirus infections for both patients and healthcare workers. Changes appeared in communication between medical staff and patients, informing, making appointments, patient management, and patient paths. To ensure safe medical care, FMPs tried to find ways to reduce the number of physical contacts between patients and medical staff to only the necessary. It was shown that in order to improve the medical care of patients with suspected Covid-19 in FMP, it was often necessary to adapt equipment, workspaces, and work process [1, 2].

In Slovenia, during Covid-19, healthcare centres were reorganized in terms of work processes and schedules; the purpose of some workspaces was changed, some personnel redistributed, communication with patients and visitors changed, protective equipment was used, triage was introduced at the entrances to healthcare centres, and certain Covid practices were opened to treat patients with suspected Covid [3, 4]. The Ministry of Health of the Republic of Slovenia created protocols for treating patients with an acute respiratory tract infection in primary care [3]. These protected medical staff and other patients from getting infected with Sars-Cov-2, but at the same time resulted in safety risks such as delays in treating patients with other medical conditions [5, 6]. This exploratory study aimed to investigate patient safety management in FMPs in Slovenia during the Covid-19 pandemic using the data from the PRICOV-19 study. The international PRICOV-19 study was led by the University of Ghent (Belgium) and involved 45 research teams from more than 35 European countries [7].

In a study published in May 2020 [8], the latest guidelines on infection prevention and control from some of the world's leading organizations and countries were reviewed: the World Health Organization (WHO), the US Center for Disease Control and Prevention (CDC), the European Center for Disease Control and Prevention (ECDC), and guidelines from Australia, the United Kingdom and China. They all emphasised the importance of administrative control with risk assessment; education, and training of healthcare workers on infection prevention and control; caution when moving patients; control of infection sources; early diagnosis and isolation of suspected cases; and a streamlined reporting system.

To our knowledge, the PRICOV-19 survey is the most comprehensive one worldwide that provides insight into the organisation of primary care during the Covid-19 pandemic. In this study we focus on five aspects of patient safety in primary care: (1) delays in care for non-Covid-19 patients due to Covid-19 protocols, (2) patient’s safety considerations made by the doctor when a referral to another medical institution was needed according to the Covid-19 protocol, (3) considerations made by the doctor regarding the feasibiliy of self-isolation at the patient’s home when this was needed according to the Covid-19 protocol; (4) efforts undertaken to safeguard safe care for vulnerable patient groups; and (5) informing community nurses when one of their patients was diagnosed with Covid-19. PRICOV-19 is also the most comprehensive study to provide insights into the practical work of family medicine residents during the Covid-19 pandemic.

## Methods

### Study design, participants and data collection

This cross-sectional study was conducted in Slovenia by the Department of Family Medicine of the University of Ljubljana in cooperation with the Department of Family Medicine of the University of Maribor. The National Medical Ethics Committee of the Republic of Slovenia approved this study on 24 September 2020 (reference number 0120–386/2020/6).

Invitations to participate were sent to the group mailing list of the Medical Chamber of Slovenia and the group mailing list of Young Doctors of Slovenia. In total 1040 Slovenian family physicians (FPs) with specialisation in family medicine, and 218 Slovenian family medicine residents were invited to participate in the study [9]. Inclusion criteria were having an active medical license, and practising medicine in a FMP. Exclusion criteria were being employed in another country than Slovenia.

Data were collected by means of an online questionnaire. The questionnaire was developed and validated at Ghent University, including a pilot study [7]. Afterwards, this was translated into Slovene using the forward–backward method [10]. Data collection in Slovenia took place from November 2020 until January 2021. The Research Electronic Data Capture (REDCap) platform was used to create, host, and distribute the online questionnaire [11]. The questionnaire was made up of fifty questions covering six domains: basic information about the doctor and FMP, patient flow, infection prevention, data management, communication with patients and cooperation, collegiality, taking care of yourself as a healthcare provider [7]. For this paper we used the questions related to the safety of patient management during the Covid-19. Data were available at three levels (Table 1): the individual participants, the FMP, and the healthcare system organization (Additional file 1).

**Table 1:** Characteristics of the participants and their practices

Characteristic	Frequency (%)
Position of participant *(n* = *191)*	
Family physician	154 (80.6)
Family medicine resident	37 (19.4)
Work experience *(n* = *191)*	
0 yrs – 5 yrs	78 (40.8)
6 yrs – 15 yrs	40 (20.9)
16 yrs – 25 yrs	38 (19.9)
> 25 yrs	35 (18.3)
Location of the practice *(n* = *190)*	
Large city	82 (43.2)
Suburbs and small towns	65 (34.2)
Mixed urban–rural	26 (13.7)
Rural	17 (8.9)
Payment system *(n* = *189)*	
Fee for service	25 (13.2)
Capitation	152 (80.4)
Other (not specified)	12 (6.3)
Family physicians' payment system* (n* = *190)*	
Salaried employment with centre of authority	141 (74.2)
Salaried employment with other family physician	26 (13.7)
Self-employed with contract(s) with health service, insurance or Authority	30 (15.8)
Self-employed without contract(s)	6 (3.2)

*Notes: yrs* - years

### Statistical analysis

Data was entered and analysed using SPSS software version 22.0. Researchers at the Ghent University was responsible for the protocol and the data cleaning [7]. For categorical variables, if there were fewer than 4 participants in a subcategory, we first attempted to merge the subcategory with another subcategory (e.g., "never" and "rarely"), and if this was not possible, the category was converted to a continuous variable. Descriptive analysis was used to describe the participants and the FMP (percentage, % and mean ± SD). A chi-square test, ANOVA, independent samples t-test or bivariate correlation test was used to explore associations between the safety of patients’ management variables and theoretically or practically used variables. A p-value lower than 0.05 was considered to be statistically significant. When a test showed a statistically significant difference between the groups, we used effect size to measure the strength of that association. For ANOVA and t-test, we used Cohen’s *d,* and the index *w* was used for the chi-square test. For Cohen’s *d* an effect size was considered to be small if *d* = 0.2, medium if *d* = 0.5, and large if *d* = 0.8. For the chi-square test, the effect size index *w* was calculated by dividing the chi-square value by the number of scores and taking the square root, and was considered small if *w* = 0.10, medium if *w* = 0.30, and large if *w* = 0.50. An effect size index represents the magnitude of an effect, independent of sample size [12]. Because of its exploratory nature this study used many tests. Therefor the Bonferroni correlations were not used.

## Results

### Sample description

In this study, 191 Slovenian respondents participated (15.2% response rate): 154 (80.6%) FPs and 37 (19.4%) family medicine residents. Response rate for the FPs with specialisation was 14,8% (154/1040) and for family medicine residents was 17% (37/218). On average the size of the FPs’ practices was 1751.8 ± 604.8 registered patients. More characteristics of the participants were shown in Table 1.

### Description of patient safety management during the Covid-19 pandemic (see Additional file 1)

Approximately half of the FPs (86, 54.8%) working in FMP reported at least once patients with a not Covid-19 related fever were seen late due to the Covid-19 protocol, and the same number (86, 54.8%) reported that patients with an urgent condition were seen late due to not coming to their FP. Approximately a quarter of FPs (40, 28.8%) reported a patient with a serious condition was seen late due to not knowing how to call their FP. Some FPs (24, 16,8%) reported a patient with a serious condition was seen late because the situation was assessed as non-urgent. A quarter of FPs reported a patient with a serious condition was seen late due to an incorrectly assessed condition (35, 27.8%).

When patients were referred to another facility, their mobility/functional status was always checked by 65.3% (111) and regularly checked by 28.2% (48) FPs. When patients needed to self-isolate, it was checked to which extent self-isolation is feasible always by 18.6% (32) and regularly by 40.7% (70) FPs.

Only one of ten FPs (17, 10.2%) drew a list from the electronic medical record for at least one group of chronic patients, yet more than half of the participants (109, 56.5%) reported that their chronic patients had been contacted for follow-up care. A quarter of FPs (44, 26.2%) contacted psychologically vulnerable patients, and only a few (9, 5.4%) contacted patients with a history of domestic violence or a problematic child-rearing situation.

Most FPs (75,3%, 122) reported that when a patient was diagnosed with Covid-19, FMP always contacted the community nurse to inform thereof. Less FPs (47,4%, 74) reported that they always contacted the community nurse when the patient was diagnosed with a major infectious disease other than Covid-19 (see Additional file 1).

### Association between aspects of patient safety management during Covid-19 pandemic and respondent and FMP characteristics (see Additional file 2)

The associations between the five aspects of patient safety management and the position of the respondent in the FMP, his/her years of experience, practice location and the practice size are described in Additional file 2. We have only found one statistically significant difference comparing reports of FPs with specialisation and family medicine residents – FPs with specialisation more often reported that at least once patient with a serious condition was seen late as compared to the family medicine residents (*p* = 0.021, *w* = 0.197) (see Additional file 2). Years of experience of FP was never statistically significant variable.

The results show that in the suburbs (including small towns) and rural environments FPs were more likely to have reported that at least once patient with a fever (not Covid-19) was seen late due to the protocol (*p* = 0.017, *w* = 0.254) and at least once patient with an urgent condition was seen late due to not coming to their FP (*p* = 0.017, *w* = 0.254). Furthermore, FPs were more likely to have reported that they had checked patients’ mobility/practical status when patients were referred to other facilities, in rural environments than in large cities, suburbs with small towns and mixed rural–urban locations. A statistically significant difference was found between rural environments and suburbs with small towns included (*p* = 0.023, *d* = 0.916).

The size of the FMP is associated with patients’ safety management during Covid-19 pandemic: the larger the FMP, the more FPs reported that at least once patient with a fever (not Covid-19) was seen late due to the protocol (*p* = 0.012, *d* = 0.415), that at least once patient with an urgent condition was seen late due to not coming to their FP (*p* = 0.012, *d* = 0.415), and that at least once patient with a serious condition was seen late due to not knowing how to call their FP (*p* = 0.025, *d* = 0.446); and when patients were referred to other facilities, their mobility/practical status was checked less frequently than at small FMPs (*p* = 0.031, *d* = 0.337).

## Discussion

Based on the results of this study, most common safety problem in Slovenian FMPs during the Covid-19 epidemic was foregone care. More delays in treatment were reported by FPs working in rural places and those working in larger FMPs.

The study demonstrates a correlation between delayed patient treatment and a FMP’s rural location in Slovenia. Association between patients with a fever (not Covid-19) seen late due to the protocol in suburbs, small towns, and rural areas has a small to medium effect size.

Similar results were found in Australia, where, in a cross-sectional study conducted among patients that contacted their FP between 1 February 2021 and 31 August 2021 due to the symptoms of a respiratory illness, found that the median number of days between the onset of symptoms of the respiratory illness until establishing contact with the FP was two days. A lower probability of coming in one day was found in rural areas and people over 65. Researchers suggest it might be a result of poorer access to medicial care in rural areas. Among the working population, they found a greater likelihood of contacting a FP within one day of the onset of symptoms, which they believed could be the result of their more frequent departure from home due to work obligations and thus the perception and also the actual greater risk of exposure to Covid-19. Unpaid sick leave and the requirement for a negative test before returning to work may also have contributed to this [13]. We found a statistically significant association in FPs reporting more often they had patients with an urgent condition seen late due to not coming to their FP concerning their location (living in the suburbs, small towns, and rural environments) with a small to medium effect size*.* Studies abroad mostly showed that patients lowered and postponed their visits to their FP during the Covid-19 pandemic due to fear of getting infected, due to campaigns encouraging patients to avoid using health services where possible [14–17], or due to exacerbated existing transportation barriers, new ones created by limitations, or outright closures of public transport [18].

Fewer FMPs in smaller towns and the countryside, and when even these are not working, the greater distance to alternative FMPs than those living in urban environments experience could be the reasons for poorer access to healthcare in rural areas in Slovenia, since as a result there are also fewer institutions for examination and/or testing of patients with suspected Sars-Cov-2 infections. These are on average more remote than in urban areas and associated with poorer public transport, if available.

The study indicates a correlation between postponed patient treatment and size of FMP in Slovenia. The associations show that the bigger size of the FMP, the more FPs reported that at least once patient with a fever (not Covid-19) was seen late due to the protocol, the more FPs reported that at least once patient with an urgent condition was seen late due to not coming to their FP and the more FPs reported that at least once patient with a serious condition was seen late due to not knowing how to call their FP (these three associations have small to medium effect size)*.*

Previous studies found limited evidence to support an association between practice size and quality of care in primary care [19]; some show that a larger group size is associated with better access and comprehensiveness but with worse continuity of care [20].

Comparing reports of FPs with specialisation and family medicine residents, we have only found one statistically significant difference – it was regarding patients with a serious condition more likely to have been seen late if reported by the FPs as compared to the family medicine residents (this association has small to medium effect size)*.*

This study supports the results of the forgone treatment of chronic patients during Covid-19 epidemic [21]. In the study, we wanted to investigate whether the Covid-19 pandemic triggered additional safety mechanisms for treating health-risk groups of patients. The study showed that approximately half of the FPs reported they contacted patients with a chronic disease (56.5%) who needed further care, and a quarter of them reported they contacted psychologically vulnerable patients (26.2%). Moreover, FPs reported that only exceptionally, they contacted patients with previous problems of domestic violence or with difficult conditions for raising children (5.4%).

Considering that FPs reported that chronic patients were significantly more contacted than those who are psychologically vulnerable and those with previous problems with domestic violence or difficult child-rearing conditions, FPs may have seen the psychiatrists and/or psychologists to whom these patients are usually referred in the role of that who would contact these patients. FPs reported that a list from the electronic file for at least one group of patients with a chronic disease (e.g., all patients who take methotrexate and need an examination) was created in 10.2% of FMPs. Even in this case, few lists could be made due to a lack of time and self-initiative, as well as due to the redeployment of nurses, who usually work and keep lists of chronic patients, to other jobs during the Covid-19 epidemic.

In Slovenia, patients suspected of being infected with Sars-Cov-2 were referred for testing to established Covid points, patients who needed an examination to special Covid practices, and patients who needed hospital care to a hospital designated for the care of Covid patients. The study showed that, when referring a patient (e.g., to a Covid point or Covid practice, to a hospital, etc.), FPs reported that the staff at FMPs almost always checked whether the patient could go there. Our study demonstrates FPs reported that patients’ mobility/practical status was on average checked more likely in rural environments with suburbs and small towns when referred to other facilities (association has large effect size) and at small practices (association has a small to medium effect size)*.*

When the patient had to self-isolate, FPs reported that the staff mostly checked whether this was feasible in the patient’s home. A possible interpretation would be that less checking of the possibility of self-isolation could be the result of the fact that FPs had no special influence on the conditions for patients’ self-isolation, but could influence the transport of patients to other medical institutions.

Countries worldwide organized primary healthcare in different ways in their fight against the Covid-19 epidemic. For example, in Hong Kong, public primary care practices were used for surveillance programs, trauma, and emergency, and patients with suspected infection were triaged to hospitals. In Singapore, public and private practices worked closely together to perform Sars-Cov-2 smears at the primary level [22].

In 2020, at the start of the pandemic, in Ireland, similar to Slovenia, primary healthcare was reorganized by establishing special Covid-19 practices, to which personal physicians referred suspected or confirmed Sars-Cov-2 infections. FPs, public health nurses, and other members of FMP teams worked in teams at such practices. They quickly developed a curriculum on infection control in the work process and the use of protective equipment. Employers sent specialists in family medicine and other primary healthcare staff to work in Covid-19 practices [23].

The Covid-19 pandemic has shed light on the importance of the health of doctors and other medical personnel and has opened up discussions on how to ensure healthcare in the future is safe for patients and health workers [2]. However, reported by FPs informing community nurses about patients suffering from Covid-19 took place more often than informing them about other important infectious diseases. This could be explained by the established notification system for Covid patients during the Covid-19 epidemic, when there were many infected people at once with a contagious disease and there were designated health workers who were in charge of notifying others about the results. But perhaps the reason lies in the fear that the Covid-19 pandemic introduced among us, with its potential for rapid spread and possible severe illness across all groups of people, including both the immunocompromised and the healthy, and even more so chronic and immunocompromised patients, and women with newborns, whom community nurses usually visit. Since community nurses make several day visits, informing them about a patient's infectious disease is extremely important.

### Practice recommendations

The smaller size of a FMP is an important factor contributing to safe patient management during the Covid-19 pandemic. In this sense, it is recommended to reduce practice size to manage patients safely, to organise the larger practices in a way that there is attention for patient safety and develop guidelines that take into account the situation of larger practices. Making access to health services more equal among urban and rural places would improve the safety of patient management in rural locations.

### Study advantages and limitations

This study's strength is that it was done during Covid-19, when research was limited due to epidemics. Thus, its results gave us precious insight into the work of family medicine specialists and residents on the front lines during the Covid-19 pandemic. Another strength is the use of an international questionnaire, developed and validated by a group of international family medicine experts.

Our study has some limitations. It was conducted online with close-ended answers, which limited the participants in answering questions. It was going on for two years and, during that time, the epidemic conditions were in flux and consequently, organizational and safety measures in primary care were constantly changing. Nevertheless, protocols regarding patients with fevers or other signs of possible Covid-19 disease remained the same in their basics – first testing for Sars-Cov-2 infections, then a medical examination, unless it was an urgent condition. Our study’s response rate was around 15%, which is a decent result for an online study, but not enough to make solid conclusions regarding the whole population of FPs in Slovenia, so results should be interpreted with care. An assessment of representativeness of the sample was made. It showed the possibility of selection bias, which means that results of this study need to be interpreted with caution [7]. This was an exploratory study and results can only serve us as guidelines to plan which variables to include in the future studies.

## Conclusion

This study showed that in Slovenia FPs reported some patients were treated with a delay during the Covid-19 pandemic in Slovenia. Care for chronic patients was problematic and neglected in certain aspects. FPs working in rural places and those working in large practices more often reported that at least once delay happened in treatment. These results should be taken into account when considering how to organize FMPs in the future. PRICOV-19 is the most comprehensive study to provide insights into the practical work of family medicine residents during the Covid-19 pandemic.

In order to increase the safety of patient management, we have to improve existing protocols for managing patients during Covid-19 and develop protocols for chronic patient management without Covid during epidemics. The results of this study may contribute to the improved preparedness of the primary healthcare system in Slovenia for future major outbreaks of infectious diseases. Further research is crucial regarding the associations between the size of practice and rural location with patients having been seen late regarding medical issues not related to Covid-19 in Slovenia.

### Supplementary Information

**Additional file 1.** The frequency of selected safety patient managements during Covid-19 epidemics as reported by FPs.

**Additional file 2.** Association between possible situations during Covid-19 and chosen variables.

## Data Availability

All data are centrally stored on the Ghent University server (Belgium). All data is anonymized at Ghent University, and all raw data that could lead to the identification of the participants is permanently removed. Researchers from partnering institutions will be able to access non-identifiable data from their national database after data cleaning. A reasonable request is required to access non-identifiable data by users who are external to the PRICOV-19 consortium. Access will be subject to a data transfer agreement and following approval from the principal investigator of the PRICOV-19 study.

## Abbreviations

CDC: The US Center for Disease Control and Prevention
Covid-19: Coronavirus disease 2019
d: Cohen's d (standard mean difference)
ECDC: European Center for Disease Control and Prevention
FMP: Family Medicine Practice
FP: Family physician
*p*-value: Probability value
REDCap: Research Electronic Data Capture
SD: Standard deviation
SPSS: Statistical Package for the Social Sciences
w: Cohen's w (square root of the standard chi-square statistic)
WHO: World Health Organization
yrs: Years
