# Supplementary material for: The safety of patient management in family medicine in Slovenia during Covid-19: a cross-sectional study
Source: BMC Prim Care. 2023 Nov 29;24(Suppl 1):255. doi: 10.1186/s12875-023-02209-z (PMC10687777; doi:10.1186/s12875-023-02209-z)
Supplement: Supplementary file 1 — Additional file 1. The frequency of selected safety patient managements during Covid-19 epidemics as reported by FPs. [file 12875_2023_2209_MOESM1_ESM.docx]

Additional file 1: The frequency of selected safety patient managements during Covid-19 epidemics as reported by FPs.

| Possible situations during Covid-19 | Frequency (%) |
| --- | --- |
| Patient with fever (not Covid-19) seen late due to the protocol (n=157) |  |
| No | 71 (45.2) |
| Yes | 86 (54.8) |
| Patient with an urgent condition seen late due to not coming to their FP (n=157) |  |
| No | 71 (45.2) |
| Yes | 86 (54.8) |
| Patient with a serious condition seen late due to not knowing how to call their FP (n=139) |  |
| No | 99 (71.2) |
| Yes | 40 (28.8) |
| Patient with a serious condition seen late because the situation was assessed as non-urgent (n=143) |  |
| No | 119 (83.2) |
| Yes | 24 (16.8) |
| Patient with a serious condition seen late due to their condition not assessed correctly (n=126) |  |
| No | 91 (72.2) |
| Yes | 35 (27.8) |
| Prepared list from electronic medical record for at least one group of chronic patients (n=166) |  |
| No | 149 (89.8) |
| Yes | 17 (10.2) |
| Chronic patients were contacted for follow-up care (n=172) |  |
| No | 63 (36.6) |
| Yes | 109 (56.5) |
| Psychologically vulnerable patients were contacted (n=168) |  |
| No | 124 (73.8) |
| Yes | 44 (26.2) |
| Patients with a history of family violence/problematic childrearing situation were contacted (n=166) |  |
| No | 157 (94.6) |
| Yes | 9 (5.4) |
| When patients are referred to another facility, their mobility/practical status is checked (n=170), mean (SD): 3.6 (0.72) |  |
| Never | 2 (1.2) |
| Rarely | 1 (0.6) |
| Sometimes | 8 (4.7) |
| Regularly | 48 (28.2) |
| Always | 111 (65.3) |
| When patients need to self-isolate, it is checked to which extent this is feasible (n=172) |  |
| Never | 17 (9.9) |
| Rarely | 27 (15.7) |
| Sometimes | 26 (15.1) |
| Regularly | 70 (40.7) |
| Always | 32 (18.6) |
| When a patient is diagnosed with Covid-19, the practice contacts a community nurse to inform them thereof (n=162) |  |
| Never | 4 (2.5) |
| Rarely | 4 (2.5) |
| Sometimes | 9 (5.6) |
| Regularly | 23 (14.2) |
| Always | 122 (75.3) |
| When a patient is diagnosed with a major infectious disease other than Covid-19, the practice contacts community nurse to inform them about it (n=162) |  |
| Never | 9 (5.8) |
| Rarely | 15 (9.6) |
| Sometimes | 18 (11.5) |
| Regularly | 40 (25.6) |
| Always | 74 (47,4) |

Notes. SD - Standard deviation. FP - Family Physician.
