# Supplementary material for: The safety of patient management in family medicine in Slovenia during Covid-19: a cross-sectional study
Source: BMC Prim Care. 2023 Nov 29;24(Suppl 1):255. doi: 10.1186/s12875-023-02209-z (PMC10687777; doi:10.1186/s12875-023-02209-z)
Supplement: Supplementary file 2 — Additional file 2. Association between possible situations during Covid-19 and chosen variables. [file 12875_2023_2209_MOESM2_ESM.docx]

*Additional file 2*: Association between possible situations during Covid-19 and chosen variables.

| Possible situations | Variables | p-value (Effect size) |
| --- | --- | --- |
| Patient with fever (not Covid-19) seen late due to the protocol (n=157) | Position in the practice | 0.315 |
|  | Years of experience | 0.149 |
|  | Location | 0.017 (*w* = 0.254) |
|  | Size of the practice | 0.012 (*d* = 0.415) |
| Patients with an urgent condition seen late due to not coming to their FP (n=157) | Position in the practice | 0.315 |
|  | Years of experience | 0.149 |
|  | Location | 0.017 (*w* = 0.254) |
|  | Size of the practice | 0.012 (*d* = 0.415) |
| Patients with a serious condition seen late due to not knowing how to call their FP (n=139) | Position in the practice | 0.021 (*w* = 0.197) |
|  | Years of experience | 0.478 |
|  | Location | 0.348 |
|  | Size of the practice | 0.025 (*d* = 0.446) |
| Patients with a serious condition seen late because the situation was assessed as non-urgent (n=143) | Position in the practice | 0.748 |
|  | Years of experience | 0.705 |
|  | Location | 0.446 |
|  | Size of the practice | 0.780 |
| Patients with a serious condition seen late due to condition not assessed correctly (n=126) | Position in the practice | 0.891 |
|  | Years of experience | 0.165 |
|  | Location | 0.309 |
|  | Size of the practice | 0.845 |
| Prepared list from electronic medical record for at least one group of chronic patients (n=166) | Position in the practice | 0.952 |
|  | Years of experience | 0.442 |
|  | Location | 0.974 |
|  | Size of the practice | 0.363 |
| Chronic patients were contacted for follow-up care (n=172) | Position in the practice | 0.459 |
|  | Years of experience | 0.907 |
|  | Location | 0.966 |
|  | Size of the practice | 0.316 |
| Psychologically vulnerable patients were contacted (n=168) | Position in the practice | 0.523 |
|  | Years of experience | 0.498 |
|  | Location | 0.486 |
|  | Size of the practice | 0.105 |
| Patients with a history of family violence/problematic child-rearing situation were contacted (n=166) | Position in the practice | 0.747 |
|  | Years of experience | 0.235 |
|  | Location | 0.566 |
|  | Size of the practice | 0.074 |
| When patients are referred to another facility, their mobility/practical status is checked (n=170) | Position in the practice | 0.062 |
|  | Years of experience | 0.403 |
|  | Location | 0.023 (*d* = 0.916) |
|  | Size of the practice | 0.031 (*d* = 0.337) |
| When patients need to self-isolate, it is checked to which extent this is feasible (n=172) | Position in the practice | 0.349 |
|  | Years of experience | 0.370 |
|  | Location | 0.055 |
|  | Size of the practice | 0.611 |
| When patients are diagnosed with Covid-19, the practice contacts the community nurse to inform them about it (n=162) | Position in the practice | 0.605 |
|  | Years of experience | 0.780 |
|  | Location | 0.556 |
|  | Size of the practice | 0.423 |
| When patients are diagnosed with a major infectious disease other than Covid-19, the practice contacts the community nurse to inform them about it (n=156) | Position in the practice | 0.719 |
|  | Years of experience | 0.810 |
|  | Location | 0.517 |
|  | Size of the practice | 0.402 |

Notes. d = cohen's d (standard mean difference); w = cohen's w (square root of the standard chi-square statistic). FP - Family Physician.
